# Case Report: Beyond two years: a neurosurgical review of prolonged survival and late recurrence in IDH-wildtype GBM

**DOI:** 10.3389/fsurg.2026.1717024

**Published:** 2026-05-11

**Authors:** Hasan Ali Aydın, Emrah Keskin, Murat Kalaycı

**Affiliations:** Faculty of Medicine, Zonguldak Bulent Ecevit University, Zonguldak, Türkiye

**Keywords:** cerebral infarction, glioblastoma multiforme, IDH-wildtype, late recurrence, neurosurgery, prolonged survival

## Abstract

IDH-wildtype glioblastoma multiforme (GBM), the most lethal primary brain tumor in adults, has a median survival of 12–15 months despite maximal multimodal therapy, including resection, radiotherapy, and chemotherapy. However, a rare subset of patients, ranging from 1% to 5%, exhibits a prognosis that defies this expectation, demonstrating prolonged survival beyond two years or late recurrence after a recurrence-free interval exceeding two years. These exceptional outcomes are shaped by tumor biology and the extent of surgical resection, underscoring the pivotal role of neurosurgery in altering GBM's relentless course. This review synthesizes current evidence on the neurosurgical strategies driving such rare successes, illuminated by a striking case of a 67-year-old female who survived 42 months with methylated O6-methylguanine-DNA methyltransferase (MGMT) and telomerase reverse transcriptase (TERT) mutation positivity following gross total resection (GTR) without neuronavigation. Enhanced by intraoperative photographs and serial MRIs, we explore the technical nuances of resection, the impact of vascular complications such as an MCA infarct, and the diagnostic challenges posed by late recurrence, including pseudoprogression. By integrating insights from literature with clinical realities, this work advocates for refined surgical approaches, including optimized resection techniques and intraoperative imaging, to improve outcomes in IDH-wildtype GBM. This approach offers neurosurgeons actionable perspectives to confront this formidable disease.

## Introduction

Glioblastoma multiforme (GBM), classified as IDH-wildtype according to the 2021 World Health Organization (WHO) criteria (1), constitutes 15% of primary brain tumors in adults, with a median survival of 12–15 months despite multimodal treatment involving maximal safe resection, radiotherapy, and temozolomide (TMZ) (2, 3). Prolonged survival beyond two years is rare, occurring in 1%–5% of patients, and is often linked to GTR and favorable molecular profiles such as methylated MGMT (4, 5). Late recurrence, defined as tumor regrowth after a period of more than two years without symptoms, is even less common and presents unique surgical and diagnostic challenges (6). Neurosurgery remains critical, yet complications like cerebral infarction can alter patient outcomes (7, 8). Neurosurgical management therefore remains central not only to cytoreduction but also to long-term outcome modulation.

This review synthesizes a neurosurgical perspective with a case study of a 67-year-old female patient who survived 42 months after GTR, complicated by an Middle cerebral artery (MCA) infarct post-second surgery (9). Supported by intraoperative photographs and serial MRIs, we draw on key studies (4, 7, 10) to explore surgical techniques, complication management, and future directions in GBM surgery (11).

## Literature review: epidemiology and surgical outcomes

The incidence of IDH-wildtype GBM is 3.19 per 100,000 individuals, with a peak occurrence among the 65–74 age group (12). The median survival period is 12–15 months, with gross-total resection (GTR) extending survival by 2–5 months compared to subtotal resection (STR) in 50%–70% of cases (13, 14). Prolonged survival has been associated with younger age, high performance status, and methylated MGMT, which enhances TMZ efficacy (15, 16). TERT promoter mutations, present in 70%–80% of IDH-wildtype GBM, correlate with aggressive biology but may influence survival in specific contexts (17, 18). Late recurrence is uncommon, attributable to dormant tumor cells or therapy-induced changes (6, 19). Postoperative infarction, defined as the occurrence of neurological impairment following tumor resection, occurs in 2%–5% of cases and has the potential to impact patient prognosis (7, 20). See Table 1 for a summary of surgical outcomes.

**Table 1:** Surgical outcomes in IDH-wildtype GBM.

Parameter	Value	Source
Incidence	3.19/100,000	(12)
Median Survival	12–15 months	(21)
Prolonged Survival	1%–5%	(4)
GTR Survival Benefit	2–5 months	(13)

Summary of incidence, median survival, proportion of patients achieving prolonged survival, and the benefit of gross-total resection (GTR) compared with subtotal resection (STR). Data highlight the survival advantage conferred by maximal resection strategies.

## Neurosurgical techniques and prolonged survival

**Figure 1:**
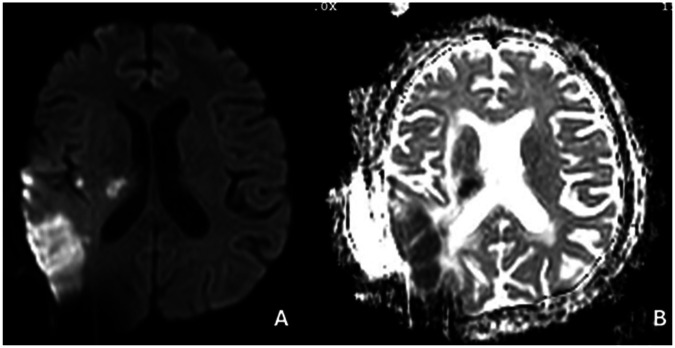
Diffusion mr image **(A,B)** after 2. Surgery right mca infarct.

**Figure 2:**
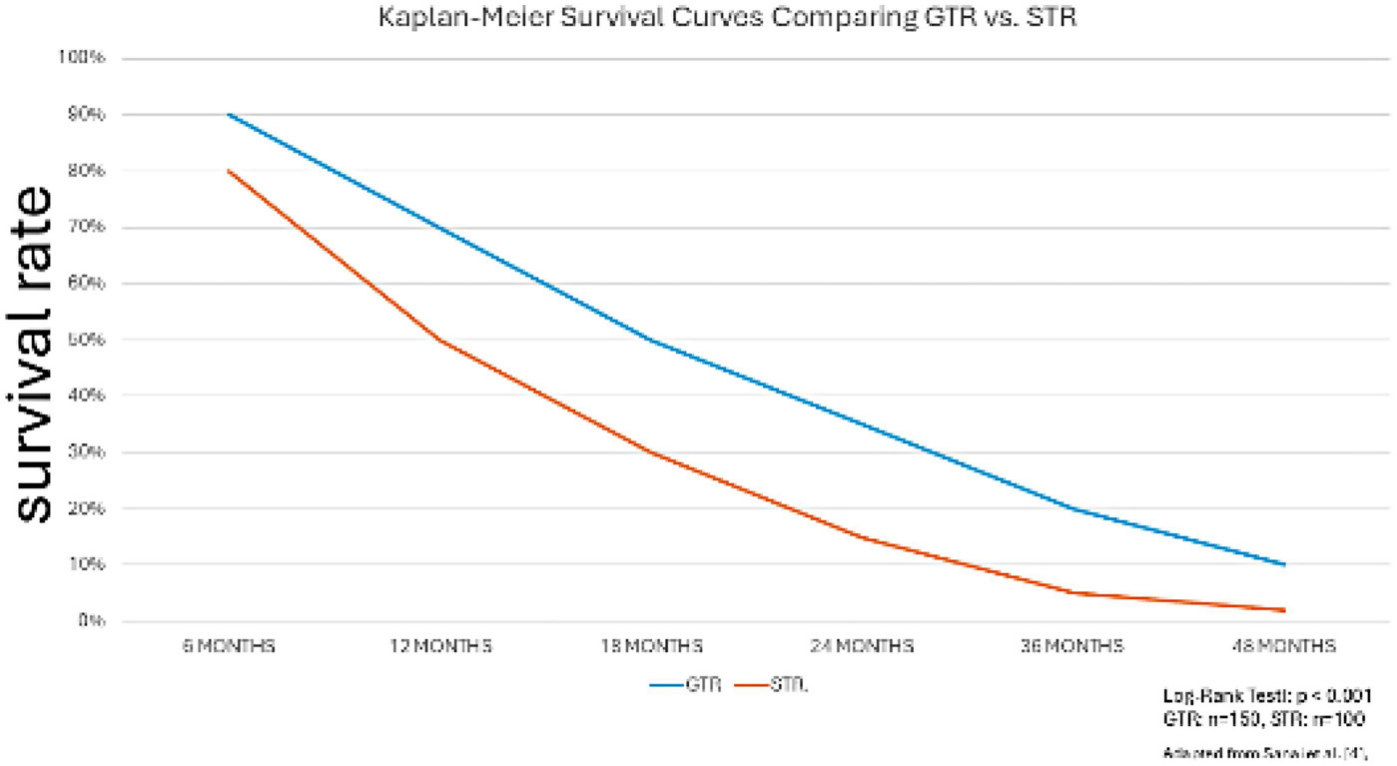
Kaplan–meier survival curves comparing gross-total resection (GTR) and subtotal resection (STR) in IDH-wildtype glioblastoma [adapted from Sanai et al. (4)]. The curves demonstrate a significant survival advantage for patients undergoing GTR. (Source: Figure 1 is a graph I drew myself.).

The principle of maximal safe resection is predicated on the delicate balance between the extirpation of neoplastic tissue and the preservation of normal structure and function (4). Gross-total resection (GTR), as confirmed by postoperative magnetic resonance imaging (e.g., Figure 1), has been shown to improve survival by 61% in comparison with sub-total resection (STR) by reducing tumor burden (13, 22). The utilization of neuronavigation techniques has been shown to reduce postoperative complications by 30%, underscoring the importance of surgical expertise in this context (7, 23). Intraoperative tools such as Doppler ultrasound offer cost-effective alternatives, reducing the risk of vascular complications (24, 25). Cerebral infarction, often near the MCA, may induce hypoxia, which could potentially delay progression (26, 27). Furthermore, the presence of methylated MGMT has been shown to enhance the response to TMZ, while TERT mutations have been observed to modulate tumor behavior (15, 28). The application of fluorescence-guided surgery has been demonstrated to further optimize resection (29). See Figure 2 for survival curves comparing GTR and STR.

## Late recurrence: surgical and diagnostic challenges

Late recurrence following a period of more than two years without symptoms is uncommon, and it is driven by quiescent tumor cells or shifts in the tumor microenvironment (6, 30). Pseudoprogression, observed in 20%–30% of cases following radiotherapy, complicates the interpretation of magnetic resonance imaging (MRI) (31, 32). Advanced imaging techniques, such as FET-PET, with a 90% specificity rate, facilitate diagnosis but require substantial resources (33, 34). Serial MRIs frequently reveal subtle changes that require surgical confirmation (35). Furthermore, the presence of methylated MGMT and TERT mutations may influence the timing of recurrence (36, 37). See Table 2 for diagnostic challenges.

**Table 2:** Diagnostic challenges in late recurrence.

Tool	Sensitivity	Specificity	Limitation	Source
Standard MRI	70%–80%	50%–60%	Pseudoprogression	(31)
FET-PET	85$–90%	90%	Cost	(33)

Comparison of diagnostic modalities for detecting late recurrence in glioblastoma. Sensitivity, specificity, and key limitations of standard MRI and FET-PET imaging are outlined, emphasizing the challenge of distinguishing true progression from pseudoprogression.

## Illustrative case

A 67-year-old female presented in August 2021 with progressive headache, mild left-sided weakness, and cognitive slowing. She had no significant family history of malignancy or known genetic syndromes. Her past medical history was notable only for well-controlled hypertension, with no prior cranial surgery or radiation exposure.

Neurological examination before the first surgery demonstrated mild left hemiparesis (Medical Research Council grade 4/5), subtle hemisensory reduction, and preserved speech. Cranial nerve examination was normal. The preoperative Karnofsky Performance Status (KPS) was 80.

**Figure 3:**
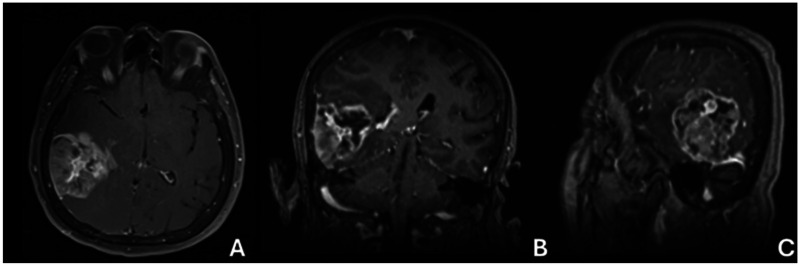
Preoperative mr image **(A)** axıal, **(B)** coronal and **(C)** sagıttal.

**Figure 4:**
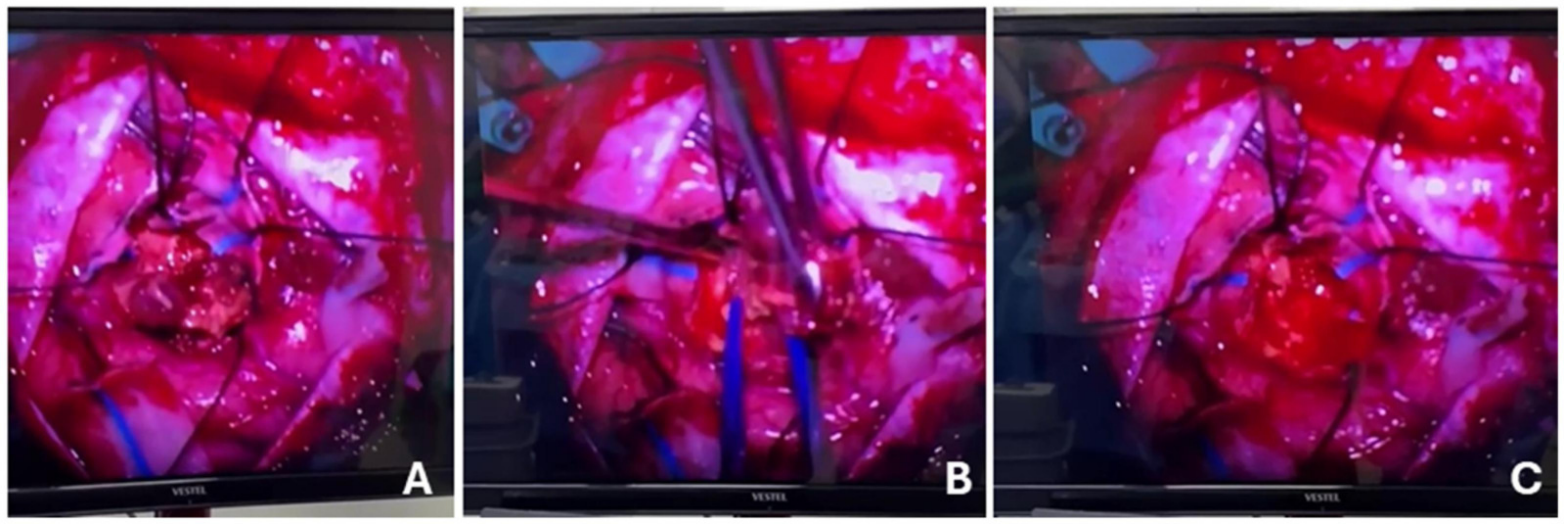
Intraoperative image **(A)** tumor exposure, **(B)** resection and **(C)** post-resection.

**Figure 5:**
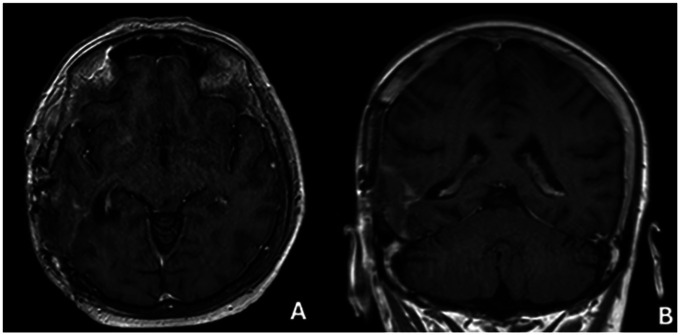
Mr image at 6 months after surgery **(A)** axıal, **(B)** coronal.

Magnetic resonance imaging revealed a right temporoparietal contrast-enhancing lesion measuring 5 × 4 × 3 cm (Figure 3). The patient underwent gross-total resection without neuronavigation (Figure 4) (38), and early postoperative MRI confirmed the absence of residual tumor (Figure 5) (39). Histopathological analysis demonstrated IDH-wildtype glioblastoma with MGMT promoter methylation, TERT mutation positivity, and a Ki-67 index of 30% (40).

**Figure 6:**
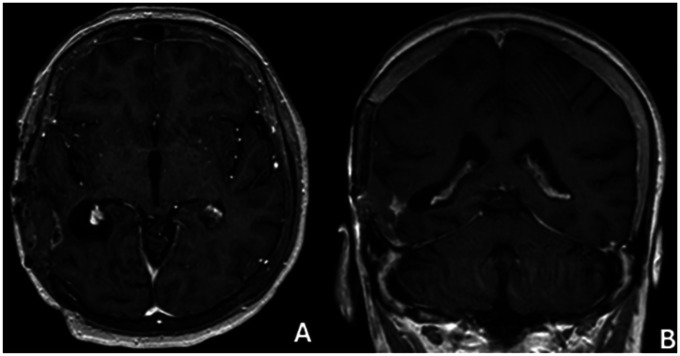
Mr image at 12 months after surgery **(A)** axıal, **(B)** coronal.

**Figure 7:**
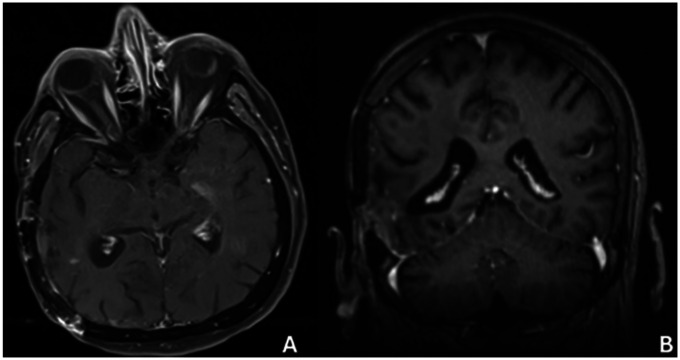
Mr image at 24 months after surgery **(A)** axıal, **(B)** coronal.

Standard adjuvant therapy consisting of 60 Gy radiotherapy with concomitant and adjuvant temozolomide was administered for six cycles (150–200 mg/m² for 5 days every 28 days). The patient remained recurrence-free for two years with stable neurological function (Figures 6, 7) (41). Follow-up MRI was performed every three months during the first two years and subsequently every six months.

**Figure 8:**
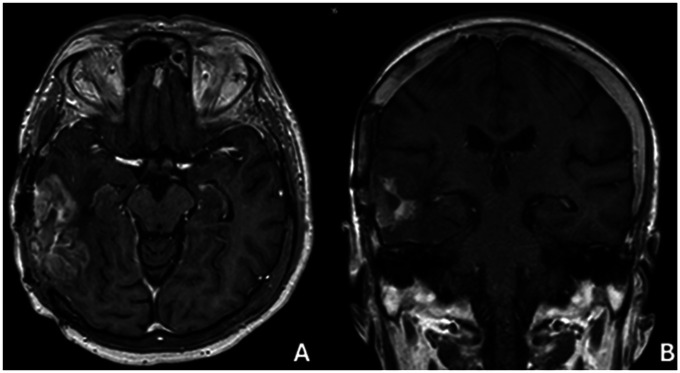
Mr image at 36 months after surgery **(A)** axıal, **(B)** coronal.

In August 2024, radiological recurrence was detected (Ki-67 7%; Figure 8) and repeat surgical resection was performed. Intraoperatively, tumor proximity to middle cerebral artery (MCA) branches and the absence of navigation increased technical complexity. Despite meticulous microsurgical dissection, postoperative imaging demonstrated an MCA territory infarction (Figure 1), resulting in neurological deterioration and a decline in KPS from 60 preoperatively to 30 postoperatively (42).

**Figure 9:**
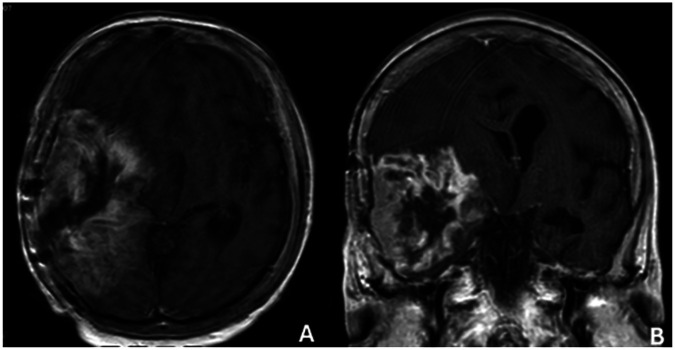
Mr image at 40 months after surgery **(A)** axıal, **(B)** coronal.

No additional chemotherapy or radiotherapy was administered after the second surgery because of poor functional status. Disease progression was documented in December 2024 (Figure 8), with further decline in KPS to 20 (43). Subsequent follow-up imaging at 40 months demonstrated extensive disease involvement of the left hemisphere with surrounding tissue changes (Figure 9). The patient remained alive at 42 months from initial diagnosis under supportive intensive care as of February 2025 (44). Ethical approval was obtained from the Zonguldak Bülent Ecevit University Ethics Committee (2025/10; 21/05/2025), and guardian consent for publication was secured (45).

Detailed neurological follow-up demonstrated progression from mild unilateral weakness to severe post-infarction hemiplegia with reduced responsiveness in the terminal phase. No systemic metastasis or secondary neurological syndromes were identified. According to family reports, the patient particularly valued the two-year period of preserved independence after initial treatment. Despite neurological decline after recurrence and infarction, prolonged survival was considered meaningful and reinforced the perceived benefit of aggressive initial surgical management.

The chronological clinical course, imaging evolution, therapeutic interventions, and functional outcomes are summarized in Table 3.

**Table 3:** Timeline of clinical course, imaging findings, and functional status.

Time Point	Imaging Findings	Treatment/Clinical Event	Neurological Status	KPS
Aug 2021	Right temporoparietal contrast-enhancing mass	First gross-total resection	Mild left hemiparesis	80
Post-op 48 h	No residual enhancing tumor	Initiation of radiotherapy + temozolomide	Stable	80
0–24 months	No recurrence on serial MRI	Completion of adjuvant therapy	Independent daily function	80–90
Aug 2024	Radiological recurrence	Second surgical resection	Moderate neurological deficit	60
Early post-op	MCA territory infarction on MRI	Supportive management only	Severe hemiplegia	30
Dec 2024	Progressive disease	Palliative follow-up	Marked neurological decline	20
Feb 2025	Persistent disease	Supportive care	Alive with severe disability	20

This table summarizes the chronological relationship between radiological findings, surgical procedures, adjuvant treatments, postoperative complications, and functional decline over the 42-month disease course.

## Discussion

This illustrative case demonstrates exceptionally prolonged survival in IDH-wildtype glioblastoma, a phenomenon observed in only a small minority of patients. Neuronavigation has been shown to enhance surgical precision and reduce complications by providing accurate intraoperative guidance; however, its availability may be limited in resource-constrained settings, thereby increasing reliance on surgical expertise. In this context, gross-total resection performed without neuronavigation proved pivotal in achieving a remarkable 42-month survival. This observation is consistent with prior literature demonstrating that maximal tumor resection reduces tumor burden and potentiates the effectiveness of adjuvant therapies such as temozolomide, particularly in the presence of MGMT promoter methylation, a key molecular predictor of prolonged survival through increased tumor sensitivity to alkylating agents (4, 15). In addition, TERT promoter mutation positivity may modulate tumor biology and recurrence dynamics, supporting emerging evidence that interactions between genetic and microenvironmental factors contribute to heterogeneous survival outcomes in IDH-wildtype GBM (17, 46).

Reported long-term survivors in IDH-wildtype glioblastoma and their principal clinical characteristics are summarized in Table 4.

**Table 4:** Reported long-term survivors in IDH-wildtype glioblastoma.

Study	Age	Molecular Features	Extent of Resection	Survival	Key Prognostic Insight
Stupp et al.	56	MGMT methylated	GTR	>24 mo	TMZ responsiveness
McGirt et al.	60	Not specified	GTR	30 mo	Extent of resection critical
Chaichana et al.	52	MGMT methylated	Multiple resections	36 mo	Repeat surgery benefit
Li et al.	48	Favorable profile	GTR	40 mo	Maximal cytoreduction
Present case	67	MGMT methylated, TERT+	GTR + reresection	**42 mo**	Combined molecular & surgical effect

Summary of representative long-term survivors reported in the literature compared with the current case.

Representative studies from the literature are summarized to highlight key prognostic determinants of extended survival, including extent of resection, molecular characteristics, and treatment responsiveness, in relation to the current illustrative patient.

The occurrence of postoperative MCA infarction following the second surgery highlights the substantial vascular risk associated with re-operative glioblastoma resection, especially in the absence of intraoperative navigation. This risk reflects the close anatomical relationship between tumor margins and critical cerebral vasculature such as MCA branches (7). Preventive strategies include meticulous microsurgical dissection with preservation of arachnoid planes, intraoperative Doppler ultrasonography for real-time vascular assessment, cautious bipolar coagulation near major vessels, and the potential use of intraoperative MRI to improve anatomical orientation and safety (24, 47). Although speculative, infarct-related hypoxia may exert paradoxical biological effects by altering the tumor microenvironment, potentially suppressing proliferation or modifying responsiveness to adjuvant therapy, a mechanism supported by studies investigating hypoxia-driven glioblastoma behavior (26, 48, 49).

Late recurrence in this case was initially obscured by dural thickening on follow-up imaging, illustrating the diagnostic limitations of conventional MRI in distinguishing treatment-related changes from true tumor progression. Such reduced specificity is particularly problematic after prolonged remission, when radiation-induced fibrosis or inflammatory alterations may mimic recurrence. Consequently, surgical exploration and histopathological confirmation remain essential for definitive diagnosis and therapeutic decision-making in selected patients (31, 45). The presence of late recurrence further underscores the biological heterogeneity of IDH-wildtype GBM and supports the concept of dormant tumor cell populations capable of delayed reactivation.

From a broader surgical perspective, prolonged survival in IDH-wildtype glioblastoma most plausibly reflects the convergence of multiple favorable determinants rather than a single dominant factor. Consistent evidence indicates that maximal safe resection, MGMT promoter methylation, preserved functional status, and sustained responsiveness to adjuvant therapy represent the most reproducible predictors of extended survival. Tumor location outside eloquent cortex may further permit aggressive cytoreduction, indirectly influencing outcome. The present case aligns with this multidimensional framework, demonstrating how surgical completeness and molecular sensitivity together may overcome the typically aggressive natural history of IDH-wildtype disease.

Management of recurrent glioblastoma requires a careful balance between oncological benefit and neurological preservation. Principles guiding re-resection include maintenance of acceptable functional status, radiologically resectable focal recurrence, realistic potential to prolong survival or improve quality of life, and integration with systemic or palliative therapies. Repeat surgery should therefore be interpreted not merely as cytoreduction, but as strategic disease modulation within a longitudinal treatment paradigm. The current case illustrates both the survival potential of re-resection and the escalating procedural risk inherent to successive operations.

Mechanistically, postoperative infarction following glioblastoma surgery may arise from direct vascular injury, arterial spasm, microthrombotic phenomena, or compromised perforator circulation in regions where tumor margins closely approximate major vessels. Re-operative surgery further increases vulnerability because of distorted anatomy, adhesions, and disrupted arachnoid planes. Although speculative, infarct-related hypoxia may also influence tumor biology through microenvironmental modulation, reinforcing the importance of meticulous microsurgical technique, intraoperative vascular assessment, and judicious risk–benefit evaluation in repeat resections.

Overall, this case integrates molecular biology, surgical strategy, vascular complication, and delayed recurrence into a single longitudinal clinical narrative. Despite the inherent limitation of a single-case design, such rare long-term survivors provide meaningful insight into the spectrum of glioblastoma behavior and offer clinically relevant lessons for neurosurgical decision-making.

## Strengths and limitations

The strengths of this report include the integration of molecular characteristics, detailed neurosurgical context, and extended longitudinal follow-up in a rare long-term survivor of IDH-wildtype glioblastoma. The combination of maximal safe resection, documented late recurrence, vascular complication, and functional outcome provides a comprehensive clinical narrative that may offer meaningful insight into the heterogeneous biological behavior of this disease.

The principal limitation is the single-case design, which inherently restricts generalizability. Nevertheless, carefully documented rare survivors remain scientifically valuable, as they can illuminate clinically relevant mechanisms of treatment response, delayed progression, and surgical risk that are not readily captured in large cohort studies.

## Conclusion

Prolonged survival beyond two years in IDH-wildtype glioblastoma remains uncommon yet clinically significant. This case underscores the central role of maximal safe resection, favorable molecular profile—including MGMT promoter methylation and TERT promoter mutation—and vigilant long-term imaging surveillance in achieving extended disease control. At the same time, it highlights the persistent risks associated with repeat surgery, particularly vascular complications such as middle cerebral artery infarction, emphasizing the delicate balance between aggressive cytoreduction and preservation of critical neurovascular structures.

Overall, the findings reflect the multifaceted challenges of glioblastoma management and reinforce the need for continued refinement of neurosurgical strategy through optimized resection techniques, judicious use of intraoperative imaging, and development of cost-effective solutions applicable to resource-limited settings. Despite its single-case nature, this report contributes meaningful clinical and biological insight into the spectrum of outcomes in IDH-wildtype glioblastoma.

## Data Availability

The original contributions presented in the study are included in the article/Supplementary Material, further inquiries can be directed to the corresponding authors.
